# Associations of psychosocial factors with pregnancy healthy life styles

**DOI:** 10.1371/journal.pone.0191723

**Published:** 2018-01-25

**Authors:** Shabnam Omidvar, Mahbobeh Faramarzi, Karimallah Hajian-Tilak, Fatemeh Nasiri Amiri

**Affiliations:** 1 Infertility and Reproductive Health Research Center, Health Research Institute, Babol University of Medical Sciences, Babol, Iran; 2 Social Determinants of Health Research Center, Health Research Institute, Babol University of Medical Sciences, Babol, Iran; 3 Cancer Research Center, Health Research Institute, Biostatistics & Epidemiology Department, Babol University of Medical Science, Babol, Iran; 4 Cellular and Molecular Biology Research Center, Health Research Institute, Babol University of Medical Sciences, Babol, Iran; University of Zurich, SWITZERLAND

## Abstract

Healthy behaviors in pregnant women have a major effect on pregnancy outcomes; however, only few studies have explored the relationship of multiple psychosocial factors with healthy lifestyles during pregnancy. The objective of this study was to investigate whether the five psychosocial factors of anxiety, stress, depression, marital dissatisfaction, and social support are associated with six domains of healthy lifestyles in pregnant women, including nutrition, physical activity, health responsibility, stress management, interpersonal relationships, and self-actualization. In this cross-sectional study, 445 pregnant women from the obstetrics clinics of the teaching hospitals of Babol University of Medical Sciences were included. The subjects answered six questionnaires, including the Health-Promoting Lifestyle Profile, Beck Depression Inventory, Prenatal Distress Questionnaire, State-Trait Anxiety Inventory, Social Support Questionnaire, and Marital Satisfaction Scale. We developed a series of simple linear regression models based on each subscale of lifestyle (nutrition, physical activity, health responsibility, stress management, interpersonal relationships, and self-actualization) as the dependent variables and the five psychological variables (anxiety, stress, depression, marital dissatisfaction, and social support) as the independent variables. State and trait anxieties were the strongest negative predictors of all aspects of a healthy lifestyle. Furthermore, depression was negatively associated with all of the six subscales of a healthy lifestyle. Pregnancy-specific stress was the only negative predictor of stress management and self-actualization. Marital dissatisfaction was negatively associated with nutrition, stress management, health responsibility, and self-actualization. Social support had negative and positive associations with healthy behaviors. The study suggests that more attention should be paid to identifying the psychological risk factors in pregnancy in addition to providing suitable interventions for improving the lifestyle of pregnant women.

## Introduction

Lifestyle is a popular concept that is often used to describe the choices people make with regard to their consumption patterns. In the context of health, these choices involve diet, cigarette smoking, alcohol consumption, and other health-related habits. [1]. Healthy lifestyles are patterns of self-initiated behaviors and perceptions that serve to maintain or enhance the level of well-being of individuals. These behaviors consist of six domains: nutrition, physical activity, health responsibility, stress management, interpersonal relationships, and self-actualization [2]. In 1995, Deluca and Lobel defined healthy and unhealthy behaviors during pregnancy by using four domains: nutrition, exercise, smoking, and substance abuse [3].

Unhealthy behaviors and lifestyles are two major causes of death worldwide [2,4]. The healthy behaviors of pregnant women affect their pregnancy outcomes. Pregnant women who are overweight or obese (body mass index >26 kg/m^2^) or women with higher weight gains during pregnancy are at a higher risk for unfavorable birth outcomes, such as pregnancy hypertension, high-birth-weight baby, preeclampsia, and emergency cesarean delivery [5]. Maternal smoking is associated with higher rates of abnormal fetal heart rate tracings during labor and higher rates of low-birth-weight babies [6]. Maconchi et al. reported that intake of supplements and eating fresh vegetables daily were risk factors for spontaneous abortion during pregnancy [7]. Furthermore, studies have shown that both stress and stress management are important factors affecting pregnancy outcomes [8–11].

The role of psychosocial factors in the healthy behaviors of pregnant women is a major issue. There is evidence supporting the presence of a relationship between healthy behaviors of pregnant women and their psychological factors. Previous studies revealed some psychological factors such as depression; stress, social support, and marital satisfaction are related to healthy behaviors in pregnant women. Depression and stress are major contributors to the healthy behaviors of pregnant women, especially in terms of physical activity, nutrition, and weight gain during pregnancy. A study reported that pregnant women with depression tend to have adverse pregnancy habits, which, in turn, have adverse effects on the outcome of pregnancy [12]. Another study emphasized that depression leads to inappropriate nutritional behaviors and low consumption of fruits [13]. There is a relationship between psychosocial distress and diet. Previous studies have shown that psychological disturbance prevents the consumption of vegetables and fruits [14,15]. Another study reported that high stress levels during pregnancy cause weight increase in pregnant women [16]. There is a correlation between body mass index and depression, which is related to weight gain during pregnancy [17]. Kim and Lee reported that women with low stress levels during pregnancy engage in more regular exercises than pregnant women with high stress levels [18]. Another study confirmed that pregnant women with lower physical activity levels had higher anxiety symptoms [19]. Social support is another important factor affecting the lifestyle of pregnant women. It is a concept that encompasses a wide range of support systems for the emotional, device, information, and evaluation aspects [20]. A review article has reported that social support has a major role in changing lifestyle managements [21]. Increasing or decreasing weight during pregnancy is associated with psychosocial factors such as pregnancy stress and social support [22]. Moreover, social support is a protective factor against pregnancy stress [23]. Findings suggest a possible relationship between marital satisfaction and lifestyle. Pregnant women who have a higher marital satisfaction have healthier and more desirable diets, and less depressive and psychological problems [24]. A weak marital relationship is the most stable predictor of anxiety and other health issues during pregnancy [25]. In addition, marital satisfaction affects the severity of depression symptoms in pregnant women [26]. Also, some previous studies have emphasized the role of some demographic factors, such as age, education, economic status, and marital status, in healthy lifestyles [21,27]

Although previous studies revealed that some psychological factors were associated with related to healthy behaviors [12–27], only few studies have explored the relationship of multiple psychosocial factors with the healthy lifestyles of pregnant women. The present study aimed to address the existing gap in the healthy lifestyle literature based on testing a model that examines the roles of five psychosocial variables on the prediction of six subscales of healthy lifestyles in pregnant women. To the authors’ knowledge, this is the first study to use the five psychosocial variables of anxiety, stress, depression, marital dissatisfaction, and social support to association with six domains of healthy lifestyles of pregnant women, including nutrition, physical activity, health responsibility, stress management, interpersonal relationships, and self-actualization.

## Methods

A cross-sectional study was conducted in four public health centers and two teaching hospitals of Babol University of Medical Sciences between January 2016 and April 2017. Singleton pregnant women who were older than 18 years, had at least 5 years of education, were willing to participate in the study, and self-reported being healthy and at a low risk of developing pregnancy complications were invited to participate in the study. The exclusion criteria were pregnancy complications (such as hypertension, diabetes, preterm labor, and maternal hemorrhage), organ defect (vision or motor), severely limited physical activity, infertility, severe medical problems during pregnancy (asthma, renal problems, thyroid problems, diabetes, preeclampsia, and intrauterine abnormalities), drug abuse and alcohol addiction, and severe psychiatric disorders (bipolar disorder, psychotic disorder, and psychiatric disorders under psychiatric treatment).

Availability sampling was utilized to recruit the pregnant women. We invited 500 women to participate in the study. Of the 445 eligible women who provided informed consent for study participation, 37 women were in early pregnancy (≤13 weeks), 200 women were in mid-pregnancy (14–26 weeks), and 208 women were in late pregnancy (27–42 weeks). Four midwives were available throughout the study to answer any possible queries. The midwives conducted an interview with the women, and obtained their medical and obstetric histories to collect demographic data and assess their obstetric risks and suitability according to the inclusion criteria. Furthermore, the midwives provided the women with a brief explanation about the purpose of the study and on how to fill in the questionnaires. The participants completed six questionnaires during their antenatal care appointments. The questionnaires included the Health-Promoting Lifestyle Profile (HPLP II), Beck Depression Inventory (BDI-II), Prenatal Distress Questionnaire (PDQ), State-Trait Anxiety Inventory (STAI), Marital Satisfaction Scale (MSS), and Social Support Questionnaire (SSQ). Ethical approval for the study was obtained from the Medical Ethics Committee of Babol University of Medical Sciences.

### Statistical analysis

Data for the descriptive analysis of all variables were presented as percentages, means, and standard deviations. Significant differences among the mean scores of variables in the three time points of pregnancy were assessed using analysis of variance (ANOVA). Correlations among continuous variables were calculated using Pearson’s test. To determine the final predictors of the six subscales of lifestyle, six psychological variables were included in two series of linear regression analyses with non-adjusted and adjusted models. We developed a series of simple linear regression models based on each subscale of lifestyle (nutrition, physical activity, health responsibility, stress management, interpersonal relationships, and self-actualization) as the dependent variables and the five psychological variables (anxiety, stress, depression, marital dissatisfaction, and social support) as the independent variables in the two-series (non-adjusted regression and adjusted regression) analysis. Age, education, and gestational age as the controlling variables were included in all the adjusted regression models. The adjusted and non-adjusted regression models were acquired from the estimated coefficients and presented with the corresponding 95% confidence intervals. A *p*-value of <0.05 was considered significant.

### Measurement

#### HPLP II

This questionnaire was designed by Walker *et al*. and contains 52 questions with responses graded on a four-part Likert scale from 1 to 4 (never, 1; sometimes, 2; often, 3; and always, 4). The scores range from 52 to 208. The healthy behaviors consist of only six domains: nutrition, physical activity, health responsibility, stress management, interpersonal relationships, and self-actualization [2]. We used the validated Persian HPLP-11 version [28].

#### PDQ

The PDQ was designed by Yali and Lobel in 1999 to assess pregnancy-specific stress. It consists of 12 items. The responses to the PDQ are graded on a three-point scale ranging from 0 (not at all) to 4 (extremely). This questionnaire has a good face validity, concurrent validity, and internal consistency. The Cronbach’s alpha coefficient was reported to be 0.81 in the second trimester of pregnancy [29]. We used the validated Persian PDQ version [30].

#### SSQ

This scale was developed by Fleming et al. in 1982 and consists of 25 items and five subscales (social support from friends, family, neighbors, and public, and opinion about such a support) [31]. The validity of the Persian SSQ was reported as 0.68 (Cronbach’s alpha level) [32].

#### STAI

Spielberger developed the STAI in 1983. This questionnaire assesses two types of anxiety: state anxiety (anxiety about an event) and trait anxiety. Subjects respond on the basis of a four-point Likert scale. The scores range from 20 to 80, with higher scores indicating greater anxieties [33]. We used the validated Persian STAI version in this study [34].

#### BDI II

This questionnaire consists of 21 questions. Each item score ranges from 0 to 3. The total scores range from 0 to 63. Higher scores indicate more severe depression symptoms [35].

#### Marital dissatisfaction

We used the short form of the MSS developed by Mehrabian in 1998 to assess marital dissatisfaction. This scale consists of 14 questions involving two factors: marital satisfaction and marital dissatisfaction. The factorial structure of the MSS confirmed the validity with an internal consistency of 0.696 (Cronbach’s alpha level) and two factors. In this study, we used seven questions related to marital dissatisfaction correlation [36]. We used the validated Persian version of the scale [37].

## Results

### Study population

Demographic characteristics of the pregnant women are shown in Table 1. Table 2 shows the comparison of the mean scores of the subscales of lifestyles based on the gestational age. The ANOVA test revealed that there was no significant difference among the mean scores of the three groups of pregnant women based on three time points (early, mid, and late pregnancy).

**Table 1:** Demographic characteristics of the population study.

Variables	
Age (years)	
≤20	21 (4.7)
21–30	338 (75.8)
≤31	87 (19.5)
Level of the education	
Primary/High school	322 (52.7)
University	211 (47.8)
Job of the women	
Unemployed	399 (89.5)
Employed	47 (10.5)
The number of the children	
0	319 (71.5)
1	106 (23.8)
≤2	21 (4.7)

**Table 2:** The mean scores of the life style and psychosocial factors of pregnant women in early, mid, and late pregnancy.

Variables	Early pregnancy	Mid pregnancy	Late pregnancy	P-value	Total population
**Life styles**					
Nutrition	2.71(0.55)	2.70 (0.48)	2.78 (0.46)	0.159	2.75 (0.47)
Physical activity	2.15 (0.66)	2.09 (0.50)	2.02 (0.55)	0.489	2.06 (0.55)
Health responsibility	2.55 (0.66)	2.55 (0.51)	2.57 (0.52)	0.731	2.56 (0.52)
Stress management	2.46 (0.60)	2.38 (0.42)	2.41 (0.45)	0.690	2.40 (0.45)
Interpersonal relationship	2.72 (0.61)	2.65 (0.48)	2.68 (0.48)	0.774	2.67 (0.51)
Self-actualization	2.69 (0.60)	2.59 (0.48)	2.71 (0.51)	0.116	2.67 (0.51)
**Anxiety**					
Stat-anxiety	41.59 (11.83)	44.02 (10.16)	41.93 (10.91)	0.175	42.59 (10.77)
Trait-anxiety	39.24 (10.42)	41.76 (10.21)	40.30 (11.24)	0.422	40.69 (10.85)
**Pregnancy Specific stress**	17.65 (9.73)	16.49 (8.01)	16.14 (8.32)	0.564	16.39 (8.34)
**Depression**	2.70 (2.47)	3.18 (4.56)	3.77 (4.62)	0.233	3.48 (4.46)
**Social support**					
Friends	2.08 (1.13)	2.16 (1.07)	2.12 (1.06)	0.865	2.13 (1.07)
Family	5.56 (1.96)	5.82 (1.41)	5.87 (1.56)	0.591	5.83 (1.55)
Neighbors	1.81 (1.34)	1.90 (1.50)	1.68 (1.38)	0.436	1.76 (1.42)
Public	3.11 (1.48)	2.69 (1.33)	2.61 (1.28)	0.179	2.68 (1.32)
Opinion about the support	3.11 (1.48)	2.69 (1.33)	2.61 (1.28)	0.004	2.68 (1.32)
**Marital dissatisfaction**	9.70 (4.22)	10.22 (3.90)	9.26 (3.82)	0.041	9.62 (3.90)

Correlation coefficient between healthy life styles and psychosocial variables are shown in Table 3. Table 4 shows the results of the series of regression analyses.

**Table 3:** Correlation coefficient between healthy life styles and psychosocial variables.

**Variables**	1	2	3	4	5	6	7	8	9	10	11	12
1.Nutrition												
2. Stress management	.399**											
3. Interpersonal Relationship	.629**	.538**										
4. Physical activity	.204**	.584**	.236**									
5. Self-actualization	584**	.642**	.755**	.357**								
6. Health responsibility	627**	.549**	.688**	.459**	.665**							
7. Total life style	.749**	.784**	.833**	.553**	.852**	.854**						
8. Pregnancy Specific stress	-.108*	-.085	-.071	.002	-.194**	-.056	-.142*					
9. Marital dissatisfaction	-.327**	-284**	-431**	-.043	-.485**	-302**	-.432**	.121*				
10. Social support	-.209**	.054	-.179**	.185**	-.113*	-.093	**-.076**	.054	.198**			
11. Stat-anxiety	-.168**	-492**	-.576**	-.161**	-.690**	-.436*8	-.634**	.288**	.480**	.174**		
12. Trait-anxiety	-.068	-315**	-389**	-.068	-.516**	-303**	-.432**	.395**	.469**	.196**	.658**	
13. Depression	-.310**	-213**	0.56	-310**	-.097	-.040	**-.063**	.197**	.024	-.219**	.195**	.273**

**P-value<0.01,

*P-value<0.05

**Table 4:** Results of adjusted* and non-adjusted linear analysis regressions for independent variables of healthy life styles in pregnant women.

Dependent variables	Independent variables	Non-adjust model			Adjust model		
		R^2^	β	Sig	R^2^	β	Sig
Nutrition Life Style	Pregnancy-specific stress	0.005	-0.068	0.177	0.016	-0.98	0.067
	Marital dissatisfaction	0.093	-0.305	<0.001	0.094	-0.308	<0.001
	State-anxiety	0.224	-0.473	<0.001	0.225	-0.471	<0.001
	Trait-anxiety	0.102	-0.319	<0.001	0.010	-0.309	<0.001
	Depression	0.002	-0.048	<0.001	0.010	-0.049	0.367
	Social Support	0.035	-0.188	<0.001	0.036	-0.177	<0.001
Stress Management	Pregnancy-specific stress	0.010	-0.0102	0.04	0.026	-0.112	0.04
	Marital dissatisfaction	0.085	-0.291	<0.001	0.122	-0.334	<0.001
	State-anxiety	0.274	-0.524	<0.001	0.276	-0.516	0.000
	Trait-anxiety	0.126	-0.360	<0.001	0.144	-0.363	0.000
	Depression	0.69	-0.263	<0.001	0.084	-0.268	0.000
	Social Support	0.002	0.048	0.36	0.017	0.060	0.287
Interpersonal relationship	Pregnancy-specific stress	0.001	-0.038	0.486	0.022	-0.047	0.411
	Marital dissatisfaction	0.153	-0.392	<0.001	0.192	-0.435	0.000
	State-anxiety	0.334	-0.578	<0.001	0.363	-0.589	0.000
	Trait-anxiety	0.121	-0.348	<0.001	0.141	-0.349	0.000
	Depression	0.005	-0.073	0.177	0.023	-0.069	0.230
	Social Support	0.020	-0.142	0.009	0.034	-0.134	0.021
Physical activity	Pregnancy-specific stress	0.001	-0.027	0.595	0.019	-0.021	0.703
	Marital dissatisfaction	0.005	-0.067	0.190	0.030	-0.109	0.053
	State-anxiety	0.052	-0.229	<0.001	0.073	-0.233	0.000
	Trait-anxiety	0.022	-0.142	0.004	0.042	-0.151	0.005
	Depression	0.065	-0.254	<0.001	0.085	-0.258	0.000
	Social Support	0.031	0.175	0.001	0.046	0.163	0.003
Self-actualization	Pregnancy-specific stress	0.033	-0.181	0.001	0.045	-0.198	<0.001
	Marital dissatisfaction	0.208	-0.456	<0.001	0.238	-0.505	<0.001
	State-anxiety	0.498	-0.070	<0.001	0.504	-0.713	<0.001
	Trait-anxiety	0.238	-0.488	<0.001	0.238	-0.486	<0.001
	Depression	0.060	-0.245	<0.001	0.077	-0.271	<0.001
	Social Support	0.005	-0.073	0.170	0.008	-0.049	0.396
Responsibility	Pregnancy-specific stress	0.001	-0.036	0.495	0.020	-0.015	0.786
	Marital dissatisfaction	0.081	-0.285	<0.001	0.127	-0.342	<0.001
	State-anxiety	0.202	-0.450	<0.001	0.211	-0.441	<0.001
	Trait-anxiety	0.085	-0.121	<0.001	0.092	-0.272	<0.001
	Depression	0.015	-0.121	0.021	0.032	-0.111	0.046
	Social Support	0.009	-0.095	0.013	0.022	-0.086	0.131

*Adjusted linear regression models with age, education, and gestational age.

### Associations of nutrition

State anxiety was the strongest negative independent variable associated with healthy nutrition of the pregnant women (β = -0.473, *p* < 0.001) of healthy nutrition in pregnant women in both the non-adjusted and adjusted regression models. Furthermore, trait anxiety (β = -0.319, *p* < 0.001), marital dissatisfaction (β = -0.305, *p* < 0.001), and social support (β = -0.188, *p* < 0.001) were significant associated negatively with healthy nutrition in both the non-adjusted and adjusted regression models. Depression was associated negatively with healthy nutrition in pregnant women in the adjusted regression mode (β = -0.177, *p* = 0.001)l. Pregnancy specific-stress was not associated significantly with healthy nutrition in both the non-adjusted and adjusted regression models (*p* < 0.005).

### Associations of stress management

State anxiety was the strongest negative independent variable associated with stress management (β = -0.524, *p* < 0.001) in pregnant women in both the non-adjusted and adjusted regression models. Moreover, trait anxiety (β = -0.360, *p* < 0.001), marital dissatisfaction (β = -0.291, *p* < 0.001), depression (β = -0.263, *p* < 0.001), and pregnancy-specific stress (β = -0.102, *p* = 0.04) were associated significantly with stress management in both the non-adjusted and adjusted regression models. Social support was not associated significantly with stress management in both the non-adjusted and adjusted regression models (*p <* 0.005).

### Associations of interpersonal relationships

State anxiety was the strongest independent variable associated with (β = -0.578, *p* < 0.001) of interpersonal relationships in pregnant women in both the non-adjusted and adjusted regression models. Furthermore, marital dissatisfaction (β = -0.392, *p* < 0.001), trait anxiety (β = -0.348, *p* < 0.001), and social support (β = -0.142, *p* < 0.05) were associated negatively with interpersonal relationships in both the non-adjusted and adjusted regression models. Pregnancy-specific stress and depression were not associated significantly with interpersonal relationships in both the non-adjusted and adjusted regression models (*p <* 0.005).

### Associations of physical activity

State anxiety was the strongest negative independent variable associated with physical activity (β = -0.229, *p* < 0.001) in pregnant women in both the non-adjusted and adjusted regression models. Furthermore, depression (β = -0.245, *p* < 0.001) and trait anxiety (β = -0.148 *p* = 0.004) were associated negatively with physical activity in both the non-adjusted and adjusted regression models. Social support was a significant positive predictor of exercise in both the non-adjusted and adjusted regression models. Pregnancy-specific stress and marital dissatisfaction were associated significantly with physical activity in both the non-adjusted and adjusted regression models (*p* < 0.005).

### Associations of self-actualization

State anxiety was the strongest negative independent variable associated with self-actualization (β = -0.705, *p* < 0.001) in pregnant women in both the non-adjusted and adjusted regression models. Moreover, trait anxiety (β = -0.488, *p* < 0.001), marital dissatisfaction (β = -0.456, *p* < 0.001), depression (β = -0.245, *p* < 0.001), and pregnancy-specific stress (β = -0.181, *p* = 0.01) were associated negatively with self-actualization in both the non-adjusted and adjusted regression models. Social support was not associated significantly with self-actualization in both the non-adjusted and adjusted regression models (*p* < 0.005).

### Associations of health responsibility

State anxiety was the strongest negative independent variable associated with health responsibility (β = -0.450, *p* < 0.001) in pregnant women in both the non-adjusted and adjusted regression models. Moreover, trait anxiety (β = -0.291, *p* < 0.001), marital dissatisfaction (β = -0.285, *p* < 0.001), and depression (β = -0.121, *p* = 0.021) were associated negatively with health responsibility in both the non-adjusted and adjusted regression models. Social support and pregnancy-specific stress were not associated significantly with health responsibility in both the non-adjusted and adjusted regression models (*p* < 0.005).

## Discussion

In this study, we included five psychological variables for determining the predictive factors of healthy lifestyle in pregnant women. The results underscore the significance of psychosocial variables for predicting healthy pregnancy lifestyles.

### Anxiety symptoms and healthy pregnancy lifestyles

The results revealed that both state anxiety and trait anxiety were the strongest negative independent variables in all domains of healthy lifestyles in pregnant women. A study showed that healthy eating index scores were significantly and negatively correlated with symptoms of depression, anxiety, and stress [38]. An earlier study also found that individuals with higher anxiety levels had lower exercise goals and engage in lesser physical activity [39]. The relationship between anxiety and physical activity is driven by anxiety-related physical sensations of activity, or may reflect the role of a more general difficulty in tolerating stress. Moreover, individuals with high levels of anxiety may be more likely to cancel plans to exercise, have decreased tendency to sustain long-term activity, and end exercise sessions in cases of negative emotional experiences.

A study reported that state and trait anxieties reduced the sense of personal responsibility in pregnant women [40]. An earlier study also found that state and trait anxieties were correlated with the type of coping mechanisms to stress during pregnancy [3]. Moreover, another study reported that the response to stress is correlated with self-actualization [41].

### Depression symptoms and healthy pregnancy lifestyles

The present study demonstrated that depression symptoms were negative associated negatively with nutrition, stress management, physical activity, health responsibility, and self-actualization. There is evidence supporting the concept that negative effects are a main negative predictor of physical activity [42]. This is similar to the results of Bae et al. [12], who concluded that pregnant women with higher depression scores had lower nutrient indices (total calcium, potassium, total folate, and plant iron levels) and lower exercise levels than pregnant women with lower depression symptoms. Furthermore, it was reported that depression was negatively correlated with five domains of lifestyle (nutrition, stress management, physical activity, health responsibility, and self-actualization) in female college students [43].

### Pregnancy-specific stress and healthy pregnancy lifestyles

The present results demonstrated that pregnancy-specific stress was associated negatively with stress management and self-actualization in pregnant women. These findings are consistent with those of another study that also reported that pregnancy-specific anxiety reduced the sense of personal responsibility in pregnant women [39]. Our finding that general anxiety was more important in predicting healthy behaviors than pregnancy-specific stress was not consistent with the results of some previous studies [44–46]. Lobe et al. reported that pregnancy-specific stress was negatively associated with unhealthy eating and positively associated with healthy behaviors such as vitamin intake and exercise. Moreover, Lobel et al. [12] concluded that pregnancy-specific stress might be a better predictor of healthy behaviors and pregnancy outcomes than general stress. This result questioned the priority of pregnancy-specific stress rather than general state anxiety in the prediction of healthy behaviors of pregnant women.

### Marital dissatisfaction and healthy pregnancy lifestyles

Our results demonstrated that marital dissatisfaction is a negative independent variable associated with nutrition, stress management, health responsibility, and self-actualization. In line with our results, an earlier study also found that marital satisfaction is correlated with the physical health status [47]. Another recent study also confirmed that marital dissatisfaction is linked to poor physical health [48]. Marital dissatisfaction was reported to be strongly correlated with depression during pregnancy [49]. Consistent with these results, a study reported that there is a relationship between marital satisfaction and self-actualization [50].

### Social support and healthy pregnancy lifestyles

The current findings revealed that social support was the only positive independent variable associated with physical activity in pregnant women. Moreover, social support was a independent variable associated with healthy nutrition and interpersonal relationships. Thornton et al. investigated the role of social support on beliefs and behavior related to weight, diet, and physical activity among pregnant women. The authors reported that informational support from husbands and some female relatives consistently influenced the participants’ motivation and beliefs about the need to link healthy behaviors to weight, diet, and physical activity [51].

These results suggest both positive and negative effects of social support on lifestyle. Although some negative social interactions may have a deleterious impact on health, most studies emphasized the positive effect of social support [23,52], whereas the negative effect of social interactions has been ignored in the literature. Recent evidence emphasizes that negative interactions can potentially be harmful. Some studies reported that negative social interactions may have more potent effects on mental health than positive interactions [53]. A study confirmed that social support has three types of influences (positive, negative, or no effect) on alcohol consumption [54]. In a meta-analysis of 28 studies on the effect of social support on well-being, Karen and Lincoln noted a lack of attention in the literature to the negative aspects of supportive relations. They recommended conducting further studies to develop assessment tools that would incorporate both positive and negative social interactions [55]. Future studies must also develop a theoretical framework for understanding the processes on how a negative social support affects healthy behaviors.

### Interpretations of the results

One explanation for the current findings is that anxiety and depression are the cardinal manifestations of emotional disturbance in psychology. Our hypothesis is that non-adherence to healthy behaviors, such as nutritional intake, physical activity, health responsibility, and self-actualization, might be features of the negative effects of anxiety, depression, or stress on the self-care of pregnant women. In individuals with high anxiety, stress, or depression levels, self-care (ability to take proper care of one own’s daily living needs, such as eating, activity, and sleeping) is diminished. Depression and anxiety predict the non-adherence to self-care. There is a relationship between depression and poorer self-care [56]. A previous study emphasized that depression may be a direct risk factor for impaired healthy behaviors through diminishing self-care in the pregnancy period [57]. Furthermore, anxiety and depression may also cause cognitive distortions that negatively affect the decision-making capacities for self-care [58]. Pregnant women with depression or anxiety symptoms may not be well nourished, experience reduced sleep, and comply poorly to prenatal care visits [59]. Moreover, pregnant women with depressive anxiety/depression symptoms and probably low self-care ability experience problems in social function [59]. Therefore, anxiety and depression have negative effects on both intra- and interpersonal relationships. Some aspects of the negative relationship between marital dissatisfaction and healthy behaviors may be related to the high mean anxiety level in the pregnant women in this study.

Although the present study had several strengths (use of five psychological predictors for six domains of lifestyle), some study features lend limitations to the analysis. First, we used an instrument to assess only healthy behaviors. The HPLP questionnaire does not evaluate impaired behaviors, such as smoking and alcohol drinking. Second, we could not conduct a continuous evaluation of the healthy behaviors during pregnancy. Therefore, we measured the variables of the pregnant women through a cross-sectional study, rather than a cohort study. Third, the study method was correlational, and cause-and-effect conclusions could not be drawn. Therefore, further studies are needed to determine empirical methods for a better understanding of the effect of stress or anxiety on healthy behaviors. The results of this study provide a foundation for future randomized control studies on improving healthy behaviors during pregnancy. Moreover, future studies could help determine whether general anxiety is a strong risk factor for healthy behaviors or whether pregnancy-specific stress is a main predictor of healthy behaviors in pregnant women.

In conclusion, the results of the present study revealed that state and trait anxieties and depression might play crucial roles in predicting all aspects of lifestyle. Pregnancy-specific stress was the only negative independent variable associated with stress management and self-actualization. Marital dissatisfaction was a negative predictor of nutrition, stress management, health responsibility, and self-actualization. Social support had negative and positive effects on healthy behaviors. Therefore, more attention should be paid to identifying psychological risk factors during pregnancy in addition to providing suitable interventions for improving the lifestyle of women during pregnancy. The psychological predictive factors determined in the present study provide additional aspects for health-care providers in developing prevention strategies to improve healthy behaviors in pregnant women.
