# Correction: Associations of psychosocial factors with pregnancy healthy life styles

**DOI:** 10.1371/journal.pone.0197389

**Published:** 2018-05-09

**Authors:** Shabnam Omidvar, Mahbobeh Faramarzi, Karimollah Hajian-Tilaki, Fatemeh Nasiri Amiri

The third author's name is spelled incorrectly. The correct name is: Karimollah Hajian-Tilaki. The correct citation is: Omidvar S, Faramarzi M, Hajian-Tilaki K, Nasiri Amiri F (2018) Associations of psychosocial factors with pregnancy healthy life styles. PLoS ONE 13(1): e0191723. https://doi.org/10.1371/journal.pone.0191723

There is an error in reference 30. The correct reference is: Faramarzi M, Pasha H. Psychometric properties of Persian version of Prenatal Distress Questionnaire. Soc Behav Pers. 2018, 45. https://doi.org/10.2224/sbp.6703. In press.
